# Identification of Metabolite Markers Associated with Kidney Function

**DOI:** 10.1155/2022/6190333

**Published:** 2022-07-26

**Authors:** Hongquan Peng, Xun Liu, Chiwa Aoieong, Tou Tou, Tsungyang Tsai, Kamleong Ngai, Hao I Cheang, Zhi Liu, Peijia Liu, Haibin Zhu

**Affiliations:** ^1^Department of Nephrology, Kiang Wu Hospital, Macau, China; ^2^Department of Nephrology, The Third Affiliated Hospital of Sun Yat-sen University, Guangzhou, China; ^3^Clinical Laboratory, Kiang Wu Hospital, Macau, China; ^4^Department of Mathematics, University of Macau, Macau, China

## Abstract

**Background:**

Chronic kidney disease (CKD) is a global public health problem. Identifying new biomarkers that can be used to calculate the glomerular filtration rate (GFR) would greatly improve the diagnosis and understanding of CKD at the molecular level. A metabolomics study of blood samples derived from patients with widely divergent glomerular filtration rates could potentially discover small molecule metabolites associated with varying kidney function.

**Methods:**

Using ultrahigh-performance liquid chromatography-tandem mass spectrometry (UPLC-MS/MS), serum was analyzed from 53 participants with a spectrum of measured GFR (by iohexol plasma clearance) ranging from normal to severe renal insufficiency. An untargeted metabolomics assay (N ¼ 214) was conducted at the Calibra-Metabolon Joint Laboratory.

**Results:**

From a large number of metabolomics-derived metabolites, the top 30 metabolites correlated to increasing renal insufficiency according to mGFR were selected by the random forest method. Significant differences in metabolite profiles with increasing stages of CKD were observed. Combining candidate lists from six other unique statistical analyses, six novel, potential metabolites that were reproducibly strongly associated with mGFR were selected, including erythronate, gulonate, C-glycosyltryptophan, N-acetylserine, N6-carbamoylthreonyladenosine, and pseudouridine. In addition, hydroxyasparagine were strongly associated with mGFR and CKD, which were unique to this study.

**Conclusions:**

Global metabolite profiling of serum yielded potentially valuable biomarkers of different stages of CKD. Additionally, these potential biomarkers might provide insight into the underlying pathophysiologic processes that contribute to the progression of CKD as well as improve GFR estimation.

## 1. Introduction

With an increasing elderly population and prevalence of obesity and diabetes, chronic kidney disease (CKD) has become a major public health concern, affecting approximately 10% of the population, posing a massive financial burden on health-care systems, and substantially increasing the risk of cardiovascular morbidity and mortality by at least 8-10 times compared to the general population [1–3]. Biomarkers offer the potential to distinguish etiologies of CKD, uncover the diagnosis at an earlier stage, and discern patients who respond to treatments from nonresponders. Creatinine is a well-established biomarker to assess kidney function [4]. However, it has limited sensitivity in the early detection of CKD [5, 6], and its use to estimate the glomerular filtration rate (GFR) [7] can be influenced by sex, age, and muscle mass. Because GFR is fundamental in assessing kidney function, and blood metabolite concentrations are known to be dependent on kidney function, a metabolomic approach to identify a metabolite signature could potentially provide remarkable insight into CKD pathogenesis and management. From a procedural perspective, a well-accepted current reference standard of measured glomerular filtration rate (mGFR) would be critical for comparison and validation. Ideally suited for this is measuring the clearance rate of the exogenous filtration marker iohexol; it is safe, straightforward, reliable, and inexpensive [8, 9].

Using a panel of filtration markers can improve precision, reduce errors caused by variation in each marker's non-GFR determinants, and decrease the need to use race and clinical characteristics as surrogates for the non-GFR determinants [10, 11]. Our study was aimed at identifying new metabolite biomarkers to optimize the measurement of GFR that perform equal to or better than creatinine.

Metabolomics is an omics technology that is a process to identify and quantitatively evaluate all small molecule metabolites among different types of biological samples such as serum, tissue, and urine. This technology is perfectly designed as a tool to discover novel glomerular filtration–related blood metabolite biomarkers that can be used in calculating the glomerular filtration rate (GFR). In the present study, GFR was measured with the plasma clearance rate of iohexol [12–14], and concurrently, estimated GFR (eGFR) was assessed based on serum creatinine and cystatin C levels [7] (a biomarker that accurately estimates GFR and reportedly can predict future risk of end-stage renal disease and death) [15]. Therefore, our metabolomic analysis of a wide range of metabolites could be correlated with mGFR to focus on potential novel filtration biomarkers with the aim of improving the estimation of GFR.

Blood metabolite levels are altered in CKD progression, prompting investigation utilizing metabolomics technologies that have led to the identification of new biomarkers [16–20]. The goal of the present study was to identify and replicate novel and known metabolites that have been reproducibly associated with mGFR and to characterize the metabolome associated with kidney function.

## 2. Materials and Methods

### 2.1. Study Participants

The study was comprised of 53 participants (19 females, 35.8%) with varying degrees of renal dysfunction. The CKD diagnosis was based on the NKF-K/DOQI guideline. The inclusion criteria were as follows: (1) age > 18 years and (2) voluntary CKD patient participation. Exclusion criteria were as follows: (1) acute kidney injury; (2) dehydration, congestive heart failure, obvious peripheral edema, and other severe fluid balance disorders; (3) physical disability and skeletal muscle atrophy; (4) urinary tract obstruction; (5) those who had recently taken the following drugs and could not suspend their use: aspirin, nonsteroidal anti-inflammatory drugs, cimetidine, or ranitidine; (6) allergy to iodine contrast agents; (7) thyroid disease; (8) pregnancy or breastfeeding; (9) cancer; and (10) dialysis. The participants were tested in a nonfasting state and received a single 5 mL infusion of iohexol (300 mg/mL, GE Healthcare, Shanghai, China), and its plasma clearance was calculated to measure GFR (mGFR) [14]. Blood samples were drawn from the contralateral upper extremity at specific time points to perform untargeted metabolomics assays (N ¼ 214) using ultrahigh-performance liquid chromatography–mass spectrometry, conducted at the Calibra-Metabolon Joint Laboratory (Hangzhou, China) using Metabolon's HD4 Discovery untargeted metabolomics platform in March 2021. The local ethics committee approved the protocol (KWH 2018-001).

### 2.2. Metabolomic Analysis

The untargeted metabolomics analysis was carried out at the Dian Calibra-Metabolon Joint Metabolomics Laboratory (Hangzhou, China). Each sample was assayed using four different UPLC-MS/MS methods. Liquid transfer was processed using a Hamilton automated MicroLab STAR® system (Hamilton, Switzerland) whenever possible. After adding a methanol-based metabolite extraction solution to each sample, the mixture was shaken vigorously for two minutes on a GenoGrinder 2010 (Spex SamplePrep, USA) shaker. Denatured proteins and other debris were removed by centrifugation. The resulting supernatant containing the extracted metabolites was aliquoted to four fractions corresponding to the four UPLC-MS/MS assays: two fractions were used for reversed-phase (RP) UPLC-MS/MS analyses in positive ion electrospray ionization (ESI) mode; one fraction was used for RP/UPLC-MS/MS analysis in negative ion ESI mode; one fraction was used for hydrophilic interaction chromatography (HILIC)/UPLC-MS/MS in negative ion ESI mode. Each fraction was dried under nitrogen gas flow and then reconstituted in a solution suited for each UPLC-MS/MS method. The raw mass spectrometry data were processed using in-house developed software. Metabolite identification was realized by matching experimental ion features to in-house library entries obtained from reference standard compounds. The three matching criteria include retention time index (RI), molecular ion mass to charge ratio (m/z), and MS/MS spectral data. For identification with high confidence, strict matching windows were applied to RI and m/z, and both the MS/MS forward and reverse matching scores between experimental data and standard compound entries were considered.

### 2.3. Statistical Methods

Values are expressed as the mean ± standard deviation (SD). Mean values and proportions were compared using one-way ANOVA and chi-square tests, respectively. A significance level of *p* < 0.05 was utilized in all tests, and SPSS-22 was used for these analyses. Principal component analysis (PCA) was conducted using R software. PCA is a dimension reduction technique that allows differences between many variables to be represented by a smaller number of variables.

A random forest was used to select metabolites that contributed the most to the group distinction. In addition, we used six well-known feature selection statistical methods, namely, least absolute shrinkage and selection operator (LASSO), optimal least absolute shrinkage and selection operator (Opt-LASSO), smoothly clipped absolute deviations (SCAD), iterative sure independent screening (ISIS), robust rank correlation-based screening (RRCS), and partial least squares (PLS), to further select the 30 most important metabolites in explaining the mGFR, which were implemented with RStudio [20].

## 3. Result

### 3.1. Demographic and Clinical Characteristics of the Study Population

Fifty-three samples were divided into four groups. The control group (normal renal function) contained 5 samples with mGFR > 90 mL/min/1.73 m^2^; the mild kidney dysfunction group contained 15 samples with 60 ≤ mGFR < 90 mL/min/1.73 m^2^; the moderate nephropathy group contained 11 samples with 30 ≤ mGFR < 60 mL/min/1.73 m^2^; and the severe nephropathy group contained 22 samples with mGFR < 30 mL/min/1.73 m^2^.

Of the 53 participants, there were 34 (64%) males, with a mean age of 49.2 ± 14.6 years (range, 18–79 years), mean height of 162.9 ± 8.2 cm (range, 148–180 cm), mean weight of 61.7 ± 9.0 kg (range, 40–78 kg), and mean body mass index of 23.2 ± 2.4 kg/m^2^ (range, 15.0 − 48.6 kg/m^2^). The mean serum creatinine was 286.7 ± 260.6 *μ*mol/L (range, 44.0–945.0 *μ*mol/L), and cystatin C was 2.47 ± 1.52 mg/L (range, 0.63-5.67 mg/L). There were no group differences in sex distribution, body mass index (BMI), diabetes, and hypertension (see Table 1).

### 3.2. Global Metabolite Determination and Significantly Altered Biochemicals

**Figure 1:**
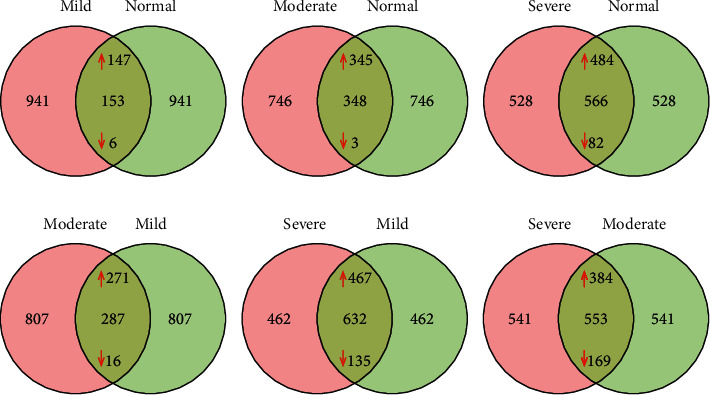
Venn diagrams to visualize the differentially expressed metabolites identified by different phenotype between the groups according to degree of renal function.

The study dataset comprised 1094 compounds with known biochemical properties. A subset of these metabolites was identified, and correlation with renal function demonstrated significant differences by CKD stage progression (*p* < 0.05). A large number of metabolites changed significantly when mGFR decreased. For example, when comparing the severe nephropathy group with the normal control group, 51.7% of the detected metabolites (566 out of 1094) changed significantly (*p* < 0.05) (Table 2). The Venn diagrams also help to visualize the differentially expressed metabolites identified by different phenotype between the groups according to degree of renal function (Figure 1).

### 3.3. High Level of Metabolite Overview

**Figure 2:**
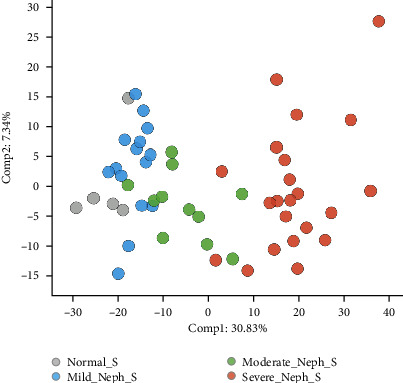
PCA of *serum* from subjects with normal kidney function (gray) and subjects with mild (blue), moderate (green), and severe (red) nephropathy.

With the detected metabolites as the variables, PCA permitted visualization of how individuals within a group cluster with respect to their data-compressed principal components.  Figure 2 shows the PCA of serum samples color-coded according to renal function grouping. There was a clear separation between the groups, best displayed along the PC1 axis, with the normal kidney function group on the left, severe nephropathy on the right, and the mild and moderate nephropathy groups in the middle; these results indicated a significantly different phenotype between the groups (Figure 2).

### 3.4. Identification of TOP Ranking Metabolite Changes

**Figure 3:**
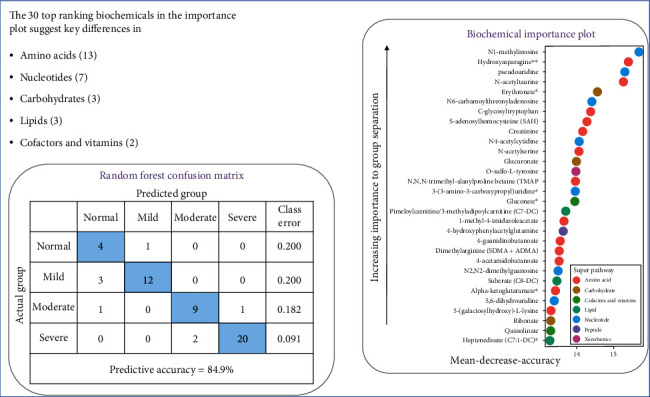
Random forest analysis of *serum* from subjects with normal kidney function, mild nephropathy, moderate nephropathy, and severe nephropathy.

In addition to producing a metric of predictive accuracy, random forest analysis also produced an associated list of biochemical rankings in order of their importance to the classification scheme. Therefore, random forest analysis was used to identify metabolites that differentiated samples from the four groups, and a predictive accuracy of 80.8% was obtained in the serum dataset (Figure 3) [21], compared to 25% by random chance alone. These results suggest that significant metabolic differences could be used to discriminate the four groups, with metabolites in the amino acid and nucleotide super pathways being of most importance for the three models.

Many metabolites related to mGFR were identified by the random forest method, which included thirteen amino acids, seven nucleotides, and three carbohydrates (Figure 3). The 30 metabolites that contributed most to the group distinction by random forest analysis included the following: N1-methylinosine (nucleotide), hydroxyasparagine (amino acid), pseudouridine (nucleotide), N-acetyltaurine (amino acid), erythronate (Carbohydrate), N6-carbamoylthreonyladenosine (nucleotide), C-glycosyltryptophan (amino acid), s-adenosylhomocysteine (SAH) (amino acid), creatinine (amino acid), N4-acetylcytidine (nucleotide), N-acetylserine (amino acid), glucuronate (Carbohydrate), O-sulfo-L-tyrosine (amino acid), N,N,N-trimethyl-alanylproline betaine (TMAP) (amino acid), 3-(3-amino-3-carboxypropyl)uridine (lipid), gulonate (cofactor and vitamins), pimeloylcarnitine/3-methyladipoylcarnitine (C7-DC) (lipid), 1-methyl-4-imidazoleacetate (amino acid), 4-hydroxyphenytacetylglutamine (peptide), 4-guanidinobutanoate (amino acid), dimethylarginine (SDMA+ADMA) (amino acid), 4-acetamidobutanoate (amino acid), N2,N2-dimethyguanosine (nucleotide), suberate (CS-DC) (lipid), alpha-ketoglutaramate (amino acid), 5,6-dihydrouridine (nucleotide), 5-(galactosylhydroxy)-L-lysine (amino acid), ribonate (carbohydrate), quinolinate (cofactor and vitamins), and heptenedioate (C7:1-DC) (lipid).

### 3.5. Six Methods for Model Selection

In addition to the random forest analysis, we used six screening methods to rank the importance of the 1094 metabolites. For each of the six statistical screening methods, we selected the top twenty markers which were the most strongly correlated with the mGFR. The top markers identified by a different method were quite different, but the first ten markers were concordantly identified by at least two methods. To further rank these top markers, we suggested two possibilities. First, we could use an integrated variable selection method. That is, the variables selected by at least k (further determined by cross validation) methods were considered as the key variables. Second, we rank the 60 variables by their Pearson correlation with the mGFR. This would seem reasonable as only a limited number of variables (60 of 1094) remained. Both are reasonable ways to provide a rank of the important markers.

**Figure 4:**
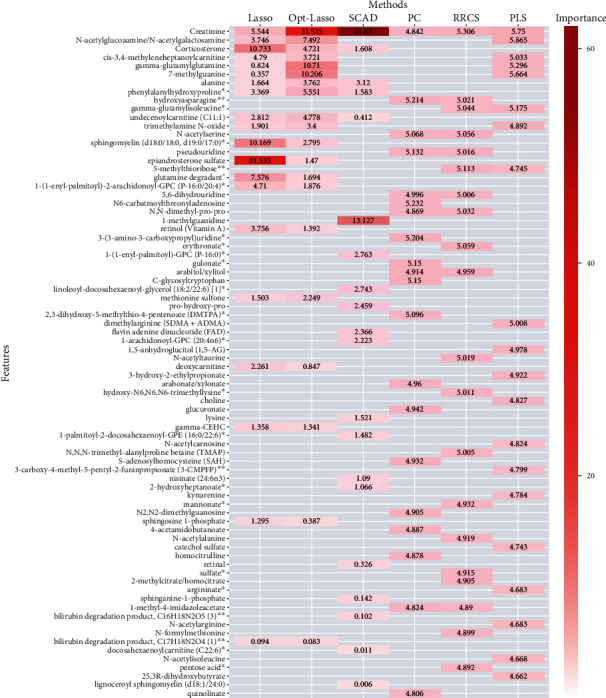
Feature selection using 6 statistical methodologies in the form of a heat plot.

Creatinine was the most important metabolite in the overall ranking derived from a weighted average of all six methods. Some other metabolites were highly correlated with kidney function, ranked from the highest down; these features were creatinine, N-acetylglucosamine/N-acetylgalactosamine, corticosterone, cis-3,4-methyl gamma-glutamylglutamine, 7-methylguanine, alanine, phenylalanylhydroxyproline, hydroxyasparagine, gamma-glutamyl-isoleucine, undecylenoyl carnitine (C11:1), trimethylamine N-oxide, N-acetylserine, sphingomyelin (d18:0/18:0, d19:0/17:0), pseudouridine, epiandrosterone sulfate, 5-methylthioribose, glutamine_degradant, 1-(1-enyl-palmitoyl)-2-arachidonoyl-GPC (P-16:0/20:4), 5,6-dihydrouridine, N6-carbamoylthreonyladenosine, N,N-dimethyl-pro-pro, 1-methylguanidine, retino (vitamin A), 3-(3-amino-3-carboxyproxypropyl)uridine, erythronate, 1-(1-enyl-palmitoyl)-GPC (P-16:0), gulonate, arabitol/xylitol, and C-glycosyltrptophan (Figure 4).

## 4. Discussion

Fundamental to the evaluation of renal function is an accurate, reliable, straightforward, relatively inexpensive method of assessing GFR. The most common laboratory tests are serum creatinine and blood urea nitrogen from which GFR is estimated. However, more accurate estimation of GFR is needed and optimally would help differentiate pathogenesis and rate of progression of CKD. In the present study, metabolomic analysis of patients with CKD revealed many metabolites linked to changes in carbohydrate, amino acid, nucleotide, and lipid metabolism. Because the course of CKD is linked to changes in metabolism, these metabolites were investigated as possible biomarkers. The identification of these potential biomarkers could aid in analyzing the various pathophysiological changes that occur in CKD, as they could indicate early abnormalities in specific pathways. Metabolomics analyses can yield hundreds to thousands of metabolites from a single sample, necessitating rapid-throughput, high sensitivity, and resolution. Recently, advances in mass methodology have allowed comprehensive studies of metabolomics and its relationship with kidney function [22–26]. Thus, the present study employed such methodologies as the heatmap and plots, principal component analysis, and random forest analysis on the entirety of the dataset. Significant differences in metabolite profiles were demonstrated in the subgroups of patients representing increasing severity of CKD. The number of biochemicals increased with CKD progression, whereas only a small number were reduced, which might indicate stage-specific biomarkers of CKD. Additionally, we found many metabolites associated with mGFR, and we analyzed the metabolites that were most strongly related to mGFR by the random forest method (Figure 3).

Sekula et al. [27] reported 56 metabolites that were associated with eGFRcr, including six that consistently showed strong correlation with eGFRcr (pseudouridine, c-mannosyltryptophan, N-acetylalanine, erythronate, myo-inositol, and N-acetylcarnosine). Moreover, Coresh et al. [16] reported a list of metabolites that could serve as a panel of filtration markers, including pseudouridine, acetylthreonine, myo-inositol, phenylacetylglutamine, and tryptophan, and a high correlation with mGFR (including all of the above metabolites except N-acetylcarnosine).

Our study identified pseudouridine and erythronate as highly correlated with mGFR, consistent with previously reported results [16, 27]. Both metabolites could be indicators of protein turnover as N-acetylation of amino acids. Pseudouridine is a derivative of uridine and is a modified nucleoside found in RNA. Importantly, pseudouridine might function as an ideal biomarker, being cited in the top 5 metabolites of the above studies; it is a stable indicator and not dependent on race.

Hydroxyasparagine were unique to the present study as biomarkers. Hydroxyasparagine, known as *β*-hydroxyasparagine (beta-hydroxyasparagine), is associated with mGFR and CKD and is a modified asparagine amino acid. However, little is known about this metabolite. It appears in posttranslational modifications of EGF-like domains that can occur in humans and other eukaryotes. The modified amino acid residue is found in fibrillin-1 [28].

In addition to searching for potential markers associated with GFR, we also investigated metabolites whose changes could be correlated with different levels of kidney function that would lend credibility to the results of our metabolomics study. Creatine kinase catalyzes the transfer of high-energy phosphate from ATP to creatine and the regeneration of ATP from creatine phosphate and ADP. In solution, creatine slowly and spontaneously cyclizes to creatinine, which is eliminated in the urine and can be used as a marker of kidney function. Creatinine has been commonly accepted as a marker of kidney filtration function. From our data, creatinine levels increased from the control group to the mild nephropathy group, the moderate nephropathy group, and the severe nephropathy group. A critical function of the kidney is to regulate electrolyte balance and fluid volume. Thus, with nephropathy, derangements in molecules necessary for osmotic regulation would be expected. As seen in Figure 3, increases in small molecules involved in osmotic regulation, such as erythronate, were observed with increasing nephropathy.

To discover and validate the novel metabolite markers related to glomerular filtration that could be used for improving eGFR, we combined the data analysis results from six different statistical screening methods, including SIS, LASSO, Optimal LASSO, SCAD, RRCS, and PLS, to determine any link between the metabolites and mGFR (Figure 4). The most frequently identified metabolites in all six methods also included several identified in the random forest (Figure 3). From our analyses, corroborated by the results reported by Coresh et al. [16] and Sekula et al. [27], the metabolites with the highest potential to measure eGFR would be erythronate, gulonate, C-glycosyltryptophan, N-acetylserine, N6-carbamoylthreonyladenosine, and pseudouridine, and hydroxyasparagine were unique to the present study as biomarkers. Worth emphasis, however, we found that creatinine was indeed included in the top 10 most important metabolites, ranking number 1 overall by the six methods we utilized and ranking number 9 by random forest.

In our study, the metabolomics results could be influenced by the type of kidney disease (e.g., inflammatory vs. noninflammatory), which makes it difficult to determine the precise cause of the differential regulation of biochemicals. However, the metabolomics study could quantitatively compare all small molecule metabolite concentrations (including well-known creatinine) based on the mGFR values and discover novel glomerular filtration–related blood biomarkers.

## 5. Conclusion

Initially, random forest analysis and six statistical models were used to identify potential glomerular filtration-related biomarkers that demonstrated a strong correlation with mGFR. Six novel, potential metabolites that were reproducibly strongly associated with mGFR were selected, including erythronate, gulonate, C-glycosyltryptophan, N-acetylserine, N6-carbamoylthreonyladenosine, and pseudouridine. In addition, hydroxyasparagine were strongly associated with mGFR and CKD, which were unique to this study. We confirmed that creatinine remained an irreplaceable biomarker of kidney function. Future studies will need to increase the number of participants to validate the biomarkers identified in this study and investigate whether our 3-5 novel biomarkers could be used individually or in combination to more accurately measure GFR.

## Figures and Tables

**Table 1:** Demographic and clinical characteristics of the study population.

Variable	mGFR ≥ 90 mL/min per 1.73 m^2^	60 ≤ mGFR < 90 mL/min per 1.73 m^2^	30 ≤ mGFR < 60 mL/min per 1.73 m^2^	mGFR < 30 mL/min per 1.73 m^2^	Overall	*p*
Sample size,	5	15	11	22	53	
Age (y)	37.2 (13.7)	42.8 (14.6)	49.0 (13.7)	56.4 (11.8)	49.2 (13.6)	0.112
Female (%)	4 (80.0)	6 (40.0)	2 (18.2)	7 (31.8)	19 (35.8)	0.693
Body mass index (kg/m^2^)	22.0 (1.1)	22.7 (2.3)	24.0 (2.1)	23.3 (2.8)	23.2 (2.5)	0.283
Diabetes prevalence (%)	2 (40.0)	6 (40.0)	6 (54.5)	9 (40.9))	23 (22.1)	0.999
Hypertension prevalence (%)	2 (60.0)	9 (60.0)	8 (72.7)	14 (63.6)	33 (62.2)	0.991
Hyperuricemia prevalence (%)	2 (40.0)	2 (13.3)	2 (18.2)	5 (22.7)	11 (20.7)	0.987
Uric acid-lowing medication use (%)	2 (40.0)	2 (13.3)	2 (18.2)	5 (22.7)	11 (20.7)	0.987
Antihypertensive medication use (%)	2 (60.0)	9 (60.0)	8 (72.7)	14 (63.6)	33 (62.2)	0.991
Glucose-lowering drug use (%)	2 (40.0)	6 (40.0)	6 (54.5)	9 (40.9))	23 (22.1)	0.999
Lipid-lowering medication use (%)	1 (20.0)	4 (26.7)	7 (63.6)	10 (45.4)	22 (41.5)	0.816
Systolic BP (mmHg)	133.6 (19.4)	138.9 (17.8)	149.1 (16.3)	142.2 (24.1)	141.9 (20.5)	0.348
Diastolic BP (mmHg)	86.9 (11.4)	85.9 (13.1)	88.9 (11.4)	85.6 (11.6)	86.9 (11.4)	0.735
Creatinine	0.6 (0.1)	1.4 (1.7)	1.8 (0.7)	5.8 (2.6)	3.2 (2.9)	<0.001
Cystatin C	0.8 (0.1)	1.3 (0.9)	1.9 (0.6)	3.9 (0.9)	2.5 (1.5)	<0.001
eGFRcr-cys (mL/min per 1.73 m^2^)	115.4 (14.9)	89.1 (26.4)	49.2 (23.4)	12.4 (7.9)	51.5 (42.2)	<0.001
mGFRcr-cys (mL/min per 1.73 m^2^)	98.3 (7.5)	73.8 (9.6)	43.5 (8.8)	14.2 (6.3)	45.1 (31.2)	

Continuous measures are summarized as the mean ± standard deviation (SD), and categorical variables are given as percentages. Values for categorical variables are given as numbers (percentages). Abbreviations: BP: blood pressure; eGFRcr-cys: estimated glomerular filtration rate, calculated by Chronic Kidney Disease Epidemiology Collaboration; mGFR: measured glomerular filtration rate. Hypertension was defined as systolic BP≧90 mmHg or 140 or diastolic BP≧90 mmHg or receiving antihypertensive medications. Diabetes was defined as fasting blood glucose≧126 mg/dL or receiving antidiabetic medications. Hyperuricemia was defined as uric acid levels≧6 mg/dL (female) and ≧ 7.0 mg/dL (male) or receiving uric acid-lowering medication.

**Table 2:** The numbers of significantly changed metabolites among different groups.

ANOVA contrasts	Mild	Moderate	Severe	Moderate	Severe	Severe
	Normal	Normal	Normal	Mild	Mild	Moderate
Total metabolites	153	348	566	287	632	553
Metabolites (↑↓)	147/6	345/3	484/82	271/16	467/165	384/169

*p* < 0.05.

## Data Availability

The datasets used to support the findings of this study are available from the corresponding author upon request.
